# Unlocking Nature’s Clock: CRISPR Technology in Flowering Time Engineering

**DOI:** 10.3390/plants12234020

**Published:** 2023-11-29

**Authors:** Ashkan Hodaei, Stefaan P. O. Werbrouck

**Affiliations:** Laboratory for Applied In Vitro Plant Biotechnology, Department of Plants and Crops, Faculty of Bioscience Engineering, Ghent University, 9000 Ghent, Belgium; ashkan.hodaei@ugent.be

**Keywords:** breeding, flowering time, genetic engineering, plant development, molecular regulation, crop improvement

## Abstract

Flowering is a crucial process in the life cycle of most plants as it is essential for the reproductive success and genetic diversity of the species. There are situations in which breeders want to expedite, delay, or prevent flowering, for example, to shorten or prolong vegetative growth, to prevent unwanted pollination, to reduce the risk of diseases or pests, or to modify the plant’s phenotypes. This review aims to provide an overview of the current state of knowledge to use CRISPR/Cas9, a powerful genome-editing technology to modify specific DNA sequences related to flowering induction. We discuss the underlying molecular mechanisms governing the regulation of the photoperiod, autonomous, vernalization, hormonal, sugar, aging, and temperature signal pathways regulating the flowering time. In addition, we are investigating the most effective strategies for nominating target genes. Furthermore, we have collected a dataset showing successful applications of CRISPR technology to accelerate flowering in several plant species from 2015 up to date. Finally, we explore the opportunities and challenges of using the potential of CRISPR technology in flowering time engineering.

## 1. Flowering Time Matters

The induction of flowering is a key process in the life cycle of mature angiosperms because it marks the transition from vegetative growth to reproductive development. During this process, shoot apical meristems (SAM) undergo several changes in their metabolism, morphology, and gene expression.

Natural selection exerts selective pressure on plants that exhibit synchronized flowering, eliminating individuals that deviate from this optimal timing. Early flowering carries risks, such as being susceptibility to damage from late frosts, and the insufficient availability of pollinators and other flowering individuals for effective pollination. Conversely, delayed flowering can lead to inhospitable conditions for seed maturation or dispersal, failure to complete the seed set before mortality from late season frost or drought, or the production of progeny in unfavorable growing environments [1,2,3,4,5].

In addition to natural selection, as part of the domestication process, early farmers made conscious or unconscious choices regarding the flowering time in light of the harvest time and yield practices. These decisions focused on strategically managing workloads over different time frames, optimizing the use of labor, and maximizing overall productivity [6,7]. Today, the flowering time is one of the main goals of breeding and genetic engineering. Longer vegetative growth leads to the development of robust vegetative organs, which in turn facilitates the development of strong reproductive organs. This, particularly in cereals such as rice, significantly increases the seed yield, quality, and nutrient accumulation [8]. In addition, precocious bolting, characterized by the early onset of flowering and seed production, generally causes some vegetables to become unusable. Addressing this problem has significant economic benefits, including extending the vegetative growing season and thus increasing yield [9,10]. In fruit trees, reducing the juvenile phase is interesting for the early production of a plantation. Breeding for late or early seasonal flowering will extend the harvest time. Alternate or biennial bearing prevents regular yields and should be avoided as it affects the yield quantity and quality [11,12,13,14]. In addition, the development of fast-flowering plant lines holds great potential for speed breeding researchers, who aim to minimize the overall duration of the breeding process, which includes various time-consuming stages of crossing, selection, and testing involved in generating new plant varieties, and can extend the timeline for developing a new variety to one or two decades [15,16].

Global climate change causes heat waves, extreme cold, changing temperatures and rainfall patterns, and disrupts the phenology of many plant species and the development of their pollinators, leading to anomalous fertility and a suboptimal fruit set [17,18,19,20,21,22,23,24,25,26]. A strategy could be to actively change the flowering time of existing elite cultivars.

The recent pioneering technique of CRISPR offers a revolutionary solution to the above challenges. To effectively select the genes to be edited, a solid understanding of the relevant pathways and molecular mechanisms is essential. In the following chapter, we will take a closer look at these mechanisms and pathways.

## 2. Molecular Mechanisms Regulating Flowering Time in Arabidopsis

A series of experiments beginning in 1865 led to the introduction of the florigen hypothesis, which postulated the existence of a substance that could be transferred from the leaf to the shoot and induce flowering. This hypothesis led to widespread research efforts to elucidate the nature and existence of florigen [27,28]. Florigen is currently considered synonymous with the flowering locus T (FT) gene, which comprises the FT mRNA, its protein, or both [28,29,30,31]. The study of FT presents challenges due to the presence of numerous paralogs, orthologs, and homologs that exhibit similar or antagonistic functional behaviors, as well as their close sequence similarity with other proteins and interactions with different pathways. For example, in the antagonistic interaction between FT and TERMINAL FLOWER 1 (TFL1), a single amino acid turns a repressor (TFL1) into an activator of flowering (FT) [32].

**Figure 1:**
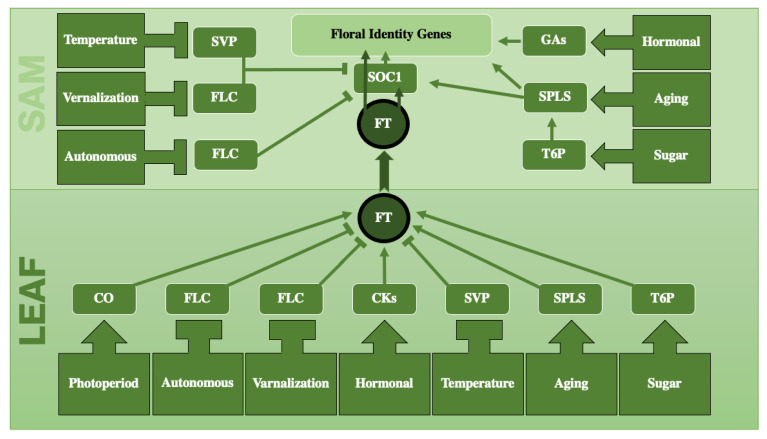
A simplified schematic representation of the physiological pathways involved in flowering, including photoperiod, autonomous, vernalization, hormonal, temperature, aging, and sugar. Each pathway has one major player which in turn regulates FT. These pathways, except for photoperiod, play a role not only in leaves, but also in shoot meristems. Flowering Locus T (FT), known as florigen, plays a central role as it travels long distances from leaf to the shoot apical meristem (SAM). Arrows indicate positive regulation, while blunt-ended arrows indicate negative regulation. Abbreviations: SVP = SHORT VEGETATIVE PHASE, FLC = FLOWERING LOCUS C, SOC1 = SUPPRESSOR OF OVEREXPRESION OF CO1, FT = FLOWERING LOCUS T, GA = GIBBERELLIC ACID, SPL = SQUAMOSA PROMOTER BINDING PROTEIN-LIKE, T6P = TREHALOSE-6-PHOSPHATE, CO = CONSTANS, CK = CYTOKININ.

The generally accepted schematic of physiological pathways proposed by Corbesier and Coupland (2005) [33] to control flowering includes four pathways: the photoperiodic, autonomous, vernalization, and gibberellin pathways. However, an updated scheme based on data collected in the Flowering Interactive Database [FLOR-ID] [34] introduces three additional pathways: sugar, aging, and temperature. Here, we present a revised figure with a simplified overview in Figure 1. Below, we explain briefly key signals or each pathway:

### 2.1. Photoperiod Pathway

The transcription factor, known as CONSTANS (CO), plays a central role in this pathway. Its role is to enhance FT expression by forming a complex with NUCLEAR FACTORY Y subunits (NF-Ys), TGACG MOTIF- BINDING FACTOR 4 (TGA4), or ASYMMETRIC LEAVES 1 (AS1). The activation of this pathway is primarily controlled by the circadian clock, orchestrated by GIGANTEA (GI), FLOWERING BHLHs (FBHs), RED AND FAR-RED INSENSITIVE 2 (RFI2), REPRESSOR OF UV-B PHOTOMORPHOGENESIS 2 (RUP2), and CONSTITUTIVE PHOTOMORPHOGENIC 1 (COP1). In addition, non-circadian regulators such as the MULTICOPY SUPPRESSOR OF IRA1 (MSI1), DAY NEUTRAL FLOWERING (DNF), and MEDIATOR25 (MED25) also play a role in the initiation of this pathway. All these elements can either positively or negatively influence the expression of CO. CO activity is also modulated by PHYTOCHROME A (PHYA), which is more sensitive to far-red light, and PHYTOCHROME B (PHYB), which responds better to red light. While PHYA acts as a positive regulator, PHYB exerts a negative control on CO. CO is regulated in transcriptional and translational levels. On short days (SDs), although the mRNA levels are high, the translation into protein does not take place. This is because COP1 and PHYB act as negative regulators during the translation process. On the other hand, on long days (LDs), GI and PHYA stimulate the translation of CO mRNA. The presence of CO protein peaks during the last hours of daylight and decreases as darkness begins [35,36,37,38,39,40,41,42,43,44,45,46].

### 2.2. Autonomous Pathway

In the autonomous pathway, the FLOWERING LOCUS C (FLC) serves as the major player, forming a complex with the SHORT VEGETATIVE PHASE (SVP) to negatively regulate FT in leaves and the SUPRESSOR OF OVEREXPRESSION OF CO1 (SOC1) in SAM.

The autonomous pathway is the most crowded of the flowering pathways, involving approximately 115 genes, mostly multifunctional, that control diverse processes including the cell cycle and DNA replication, chromatin modification, transcriptional regulation, the control of protein stability, and the processing of mRNA and microRNA. Notably, FLOWERING CONTROL LOCUS A (FCA) induces a reduction in FLC mRNA levels, FLOWERING LOCUS D (FLD) represses FLC by facilitating histone H3 Lys-4 demethylation at the FLC site, and FLOWERING LOCUS KH (FLK) represses FLC through post-transcriptional modification. It is also important to note that biotic stress, which affects flowering, is also considered as a part of the autonomous pathway. For instance, a single mutation in CADMIUM SENSITIVE 2 (CAD2), which is responsible for the biotic stress response, shows a delayed flowering phenotype under long days in Arabidopsis [47,48,49].

### 2.3. Vernalization Pathway

The vernalization pathway has FLC as its central component, similar to the autonomous pathway. Before the cold phase, six protein complexes (FRIGIDA, COMPASS, RAD6-BRE1, RAF1, SWA1, and FACT) are more active and positively regulate FLC in the transcriptional level. After cold exposure, three different protein complexes (PRC2, PRC1-like, and HDAC) increase their role in repressing FLC by not allowing RNA Polymerase to attach to the FLC locus. The prolonged exposure to cold shifts more cells to suppress FLC, increasing FT signaling from the leaves to SAM, and increases SOC1 activity in the SAM and, therefore, activates the meristem identity genes (MIs) to facilitate the transition from the vegetative to the reproductive phase [50,51,52,53,54].

### 2.4. Hormonal Pathway

The hormonal pathway, also known as the gibberellin pathway, involves the activity of cytokinins and gibberellins. Cytokinin affects the TWIN SISTER OF FT (TSF), while GAs affect the regulation of FT. Cytokinin, in a putative mechanism, positively regulates the transcription of TSF both in leaves and in SAM. TSF can be transferred from the leaf to the SAM and forms a complex there with a transcription factor, FD, and acts directly on MIs, making this the only pathway that can affect flowering independently of FT and SOC1.

In addition, GA4 boosts GID proteins, resulting in reduced DELLA proteins, which subsequently downregulate FT in leaves and MIs in SAM. In essence, GA4 positively controls FT in leaves and MIs in SAM, but negatively regulates SOC1 via its interaction with SQUAMOSA PROMOTER BINDING PROTEIN-LIKE 9 (SPL9).

Gibberellin signaling affects flowering by interfering with established endogenous and environmental flowering pathways, as well as by interacting with various phytohormone signaling pathways. In addition, cytokinins (CKs), abscisic acid (ABA), jasmonic acid (JA), ethylene (ET), brassinosteroids (BRs), and auxin also interact with DELLA proteins and, therefore, affect MIs. In conclusion, although this pathway has been traditionally referred to as the “Gibberellic pathway”, it would be more accurate to call it the “Hormonal pathway” [55,56,57,58,59].

### 2.5. Sugar Pathway

Trehalose-6-phosphate (T6P) is the main actor of this pathway which positively regulates FT in leaves and SOC1 in SAM by affecting the MIR156-SPL9 pathway. This signaling molecule (T6P) is the product of URACIL-DIPHOSPHATE GLUCOSE (UDPG) and GLUCOSE 6-PHOSPHATE (G6P) catalyzed by TREHALOSE-6-PHOSPHATE SYNTHASE 1 (TPS1). It is highly dependent on photosynthesis and reaches its peak concentration just before darkness under LD conditions, gradually decreasing with the onset of darkness [60,61,62,63,64,65]. In addition to being affected by the sugar pathway, FT also has an effect on sugar transport by activating sugar transporters such as SWEET10 [66].

### 2.6. Aging Pathway

The aging pathway, delays flowering in the juvenile phase and promotes it in the adult phase, even in the absence of external triggers. In particular, the miR156, SPL transcription factors (SPLs), miR172, SCHLAFMUTZE (SMZ), and TARGET OF EARLY ACTIVATION TAGGED 1 (TOE1) are key players in this ageing pathway, driving the transition from the juvenile to adult phase and then promoting flowering. MiRNA156 is more abundant in young plants and decreases as they mature, while miRNA172 shows the opposite pattern, increasing with age. The SMZ and TOE1 exert a repressive influence on the FT gene in the leaves and APETALA1 (AP1) in the SAM, affecting the expression of MIs and, consequently, controlling the initiation of flowering [67,68,69,70,71,72,73,74].

### 2.7. Temperature Pathway

The ambient temperature pathway influences FT through various components. At high temperatures, PHYTOCHROME INTERACTING FACTOR 4 (PIF4) and FLOWERING CONTROL LOCUS A (FCA) enhance FT. Conversely, at low temperatures, the SHORT VEGETATIVE PHASE (SVP) negatively regulates FT and SOC1 through a complex with FLC (a product of the vernalization pathway) [75,76,77,78,79,80,81,82,83,84,85,86].

### 2.8. Interconnectedness between Pathways

It is important to note that these pathways interact with each other, and it is difficult to completely distinguish between them. For example, miR172 is influenced by SPLs (“aging pathway”), FCA (“temperature pathway”), and GI (“Photoperiodic pathway”), demonstrating the interconnectedness of these pathways. Or in another example, the production of T6P, which is considered as the key player of the sugar pathway, is obviously dependent on photosynthesis and, therefore, the photoperiodic pathway.

### 2.9. Transportation of FT

FT and its homologous counterparts, known as FT-likes (FTLs), are synthesized in leaf tissues via the above-mentioned biochemical pathways. These molecules are then transported by specific transporters to the apical meristems in the shoots. The process of FT migration, a 22 kD protein with 175 amino acids, can be divided into two steps: first, it must be exported from companion cells into sieve elements, and second, it must cross sieve elements to reach the SAM.

The involvement of the transmembrane region proteins FT-INTERACTING PROTEIN1 (FTIP1) and QUIRKY (QKY) in the initial export step has been identified. To reach the SAM, another protein called SODIUM POTASSIUM ROOTDEFECTIVE 1 (NaKR1) with a heavy-metal-associated (HMA) domain plays a crucial role. The activity of NaKR1 is positively regulated by the product of the photoperiodic pathway, CO, in the model plant Arabidopsis [30,87,88,89].

In rice, the long-distance transporter for FT is the tetratricopeptide repeat 075 (TPR075) protein, while FTIP1 and FTIP9 are involved in the translocation of FT from companion cells to sieve elements [90].

Taken together, the FTIPs appear to play a critical role in the short-range transport of FT. Given recent research suggesting the involvement of different proteins in the long-distance transport of FT, further investigation is needed to determine whether the ortholog of TPR075 has a similar function in dicots, whether the ortholog of NaKR1 plays a role in monocots, and whether other heavy-metal-associated domain proteins or tetratricopeptide repeat proteins may also play a role.

In summary, our understanding of florigen transport remains largely incomplete, highlighting the need for comprehensive studies in a wide range of plant species to develop a more comprehensive understanding.

## 3. Optimal Gene Targeting Strategy for Flowering Time Engineering

### 3.1. Altering Flowering Time

Identifying the most effective gene(s) for either delaying or accelerating the flowering process depends on variables such as the species, cultivar, and growth environment, such as the plant’s response to the day length. The first step is to identify the predominant pathway(s) involved. For example, in the case of cabbage, where vernalization is a key determinant, genes such as BraFLC2, BraFLC3, AGL19s, and AGL24s associated with the vernalization pathway have shown remarkable success in breeding trials [91,92].

However, as a comprehensive strategy, targeting key proteins that are central to the overall flowering mechanism is a viable option. In genes such as FT, TFL1, and SOC1, as detailed in Table 1, 20% of efforts were by knock outing these genes. FT affects the expression of approximately 3652 genes [93]. However, from a breeding perspective, it does not appear to pose a significant challenge to the breeding objectives, except for its impact on seed dormancy [94,95]. In addition, FT affects the expression of some sugar transporters, suggesting a potential influence on other sugar-related traits. However, there is a paucity of research investigating the relationship between FT and traits such as fruit flavor, which warrants further investigation. Putting all this together, the modulation of the FT function is unlikely to result in major abnormalities. Conversely, the manipulation of TFL1, a flowering repressor, could be challenging, as some studies suggest that TFL1 mutations can lead to the aberrant development of floral structures [95,96,97,98,99]. To achieve the precise control of flowering, it may be prudent to explore alternative regulators of the FT gene, taking into account the primary pathway in your specific plant that effects greater FT expression. Alternatively, the modification of FT(s)’s Cis-regulatory elements can be used to precisely control the timing and level of expression too.

In addition to TFL1, in Arabidopsis, AGAMOUS-LIKE 12 (AGL12) displays late flowering under LD, but also the short root phenotype [100]. The overexpression of C-REPEAT/DRE BINDING FACTOR 1 (CBF1) results in late flowering under LD, but also a reduction in the freezing tolerance [101]. CURVY 1 (CVY1) single mutant flowers early under SD; however, as a side effect, altered trichome development and an increased number of siliques were observed [102]. AGAMOUS-LIKE 19 (AGL19) single mutant shows slightly late flowering under a short day, but also reduced the sensibility to vernalization [103].

**Figure 2:**
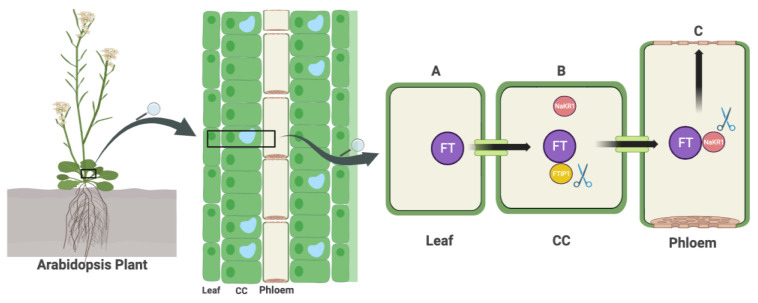
The transport of FT from the leaf to the SAM involves a number of steps. First, FT is synthesized in the leaf through the pathways shown in Figure 1 (**A**). Next, FTIP1 facilitates the transfer of FT from CC to SE (**B**). Finally, NaKR1 is responsible for the long-distance transport of FT into the SAM (**C**). The scissor symbols in the diagram indicate potential CRISPR knockout sites that could be used to disrupt FT transport. Abbreviations: FT = FLOWERING LOCUS T, SAM = SHOOT APICAL MERISTEM, FTIP1 = FT-INTERACTION PROTEIN 1, CC = Companion cell, SE = Sieve element, NaKR1 = SODIUM POTASSIUM ROOT DEFECTIVE 1 (Created with BioRender.com).

The concept of interfering with transporters has also become a subject of debate. First, it is likely that NaKR1 in Arabidopsis or TPR075 in rice are not the only transporters of the FT protein. In addition, FT mRNA has the ability to migrate as well. Furthermore, NaKR1 also serves as a transporter for a wide range of sugars [89]. Mutations in NaKR1 could lead to variations in the concentration of both primary and secondary metabolites (Figure 2).

From a holistic perspective, a breeding project focusing on flowering time requires us to consider the various interrelated pathways highlighted in this article, including signaling proteins, miRNAs, transcription regulators, transporters, etc. By recognizing the importance of these key components, we can gain a thorough understanding of the complex mechanisms that regulate flowering time and, therefore, successful breeding.

**Figure 3:**
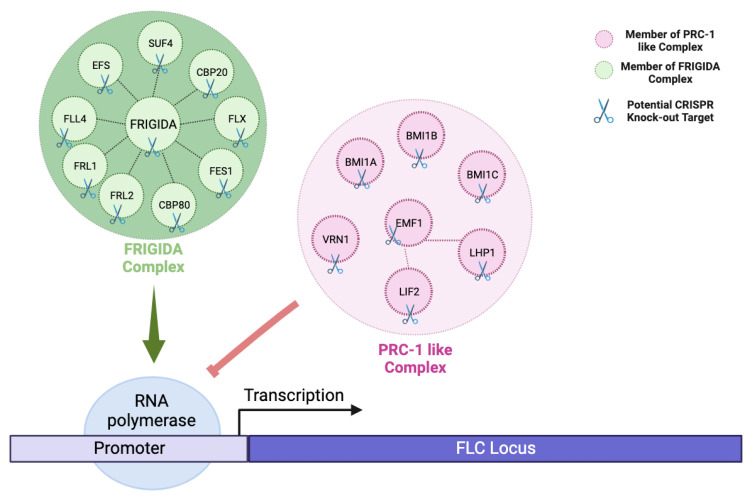
Showing the effect of cis-regulatory elements in controlling gene expression at the transcriptional level in CRISPR-based flowering time engineering. By highlighting key regulators such as FRIGIDA and PRC-1-like complexes, the figure illustrates their ability to fine-tune FLC expression either positively or negatively in transcriptional regulation. Green arrow indicates as positive regulation, while red blunt-ended arrow indicates as negative regulation. Abbreviations: SUF4 = SUPRESSOR OF FRIGIDA 4, CBP20 = CAP-BINDING PROTEIN 20, FLX = FLC EXPRESSOR, FES1 = FRIGIDA ESSENTIAL 1, CBP80 = CAP BINDING PROTEIN 80, FRL2 = FRIGIDA LIKE 2, FRL1 = FRIGIDA LIKE 1, FLL4 = FLOWERING LOCUS C EXPRESSOR-LIKE 4, EFS = EARLY FLOWERING IN SHORT DAYS, BMI1A = DREB2A-INTERACTING PROTEIN 2, BMI1B = DREB2A-INTERACTING PROTEIN 1, BMI1C = BMI1C, VRN1 = REDUCED VERNALIZATION RESPONSE 1, EMF1 = EMBRYONIC FLOWER 1, LHP1 = LIKE HETEROCHROMATIN PROTEIN 1, LIF2 = LHP1-INTERACTING FACTOR [31] (Created with BioRender.com).

As many of the proteins involved in flowering have multiple functions and diverse effects, one strategy for regulating their activity is to place greater emphasis on manipulating cis-regulatory elements (Figure 3). This approach allows us not only to fine-tune the level of gene expression, but also to precisely control the timing and location of expression. This is exactly what happened during the domestication process. To illustrate, in Brassica napus, a mutation in the transcription factor BnFLC.A10 was key to successfully altering the timing of flowering [104]. Another example is found in maize, where mutations in the transcription factors CCT and Vgt1/Rap2.7 led to significant adjustments in the flowering time [105,106].

As recent advancements, in grapefruit plants, CsLOB1 knockout mutants by CRISPR showed a delayed flowering time compared to WT [107]. The technique of dCas9 SunTag actively generated DNA methylation at the FWA promoter region, resulting in promoter silencing and the early flowering phenotype in Arabidopsis [108]. In Arabidopsis, the MS2-p300 CRISPR/dCas9 system, which incorporates H3K27 acetyltransferase as an effector domain and is linked to a nuclear-targeted MS2, has been used to modify the promoter region of the Flowering locus T (FT) gene. This resulted in a substantial two-fold increase in H3K27 acetylation within the FT promoter, which subsequently led to a significant alteration in the flowering time [109].

### 3.2. Stop Flowering

The idea of non-flowering angiosperms seems unlikely from a botanical point of view. However, in horticulture, postponing the flowering process could lead to a state of non-flowering cultivars. The disruption of FT or its transporter alone is not sufficient to achieve this result. This is due to the probability that FT is not solely dependent on a single transporter, and also because FT is not the only signaling molecule transmitted from the leaves to the shoot apical meristem (SAM). In fact, there are at least three known molecules—TSF, T6P, and AGL17—that also transduce the signal. What is more, certain pathways in the SAM can function independently of the leaves, ultimately influencing the expression of MI genes and thus the development of floral structures.

Of particular note is the structural similarity between FT and TFL1, two proteins with opposing functions. By exploiting these similarities, we can change specific amino acids, such as substituting His-88 in TFL1 and Tyr-85 in FT [32]. This simple amino acid change, with only a single nucleotide difference between His and Tyr, can switch their roles between repressor and activator. This insight holds considerable promise for targeted CRISPR breeding programs aimed at manipulating the flowering time, for example, by strategically designing gRNA sequences to introduce such mutations using base or prime editing.

## 4. CRISPR-Mediated Modulation of Flowering Time in Literature

**Figure 4:**
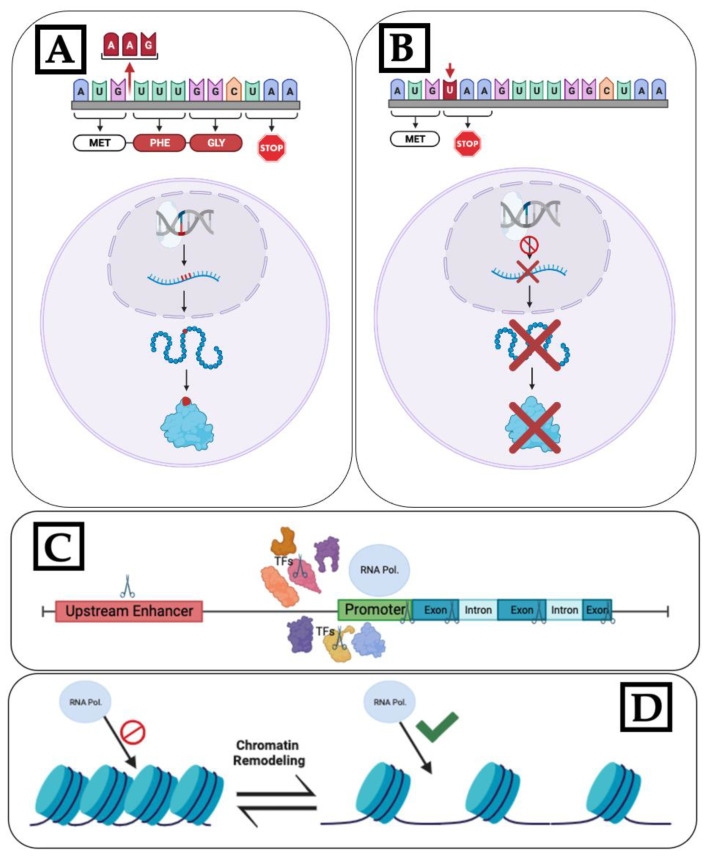
Various CRISPR-Cas approaches to modulate flowering time: (**A**) Cas cleavage-induced amino acid changes resulting in non-functional protein. (**B**) Cas cleavage resulting in stop codon insertion, terminating transcription, and preventing mRNA and, therefore, protein production. (**C**) Disruption of gene transcription by targeting elements involved in transcription, such as transcription factors, promoters, and upstream enhancer regions. The scissor symbols in the diagram indicate potential CRISPR knockout targets (**D**) Genes that regulate chromatin remodeling act as activators or inhibitors, affecting transcription by controlling chromatin structure (Created with BioRender.com).

Since 2015, there has been a striking trend in the scientific literature regarding the manipulation of the flowering time using CRISPR technology. From then until now (mid-2023), there have been numerous publications investigating different gene families. These studies cover a wide range of plant species, with a particular focus on altering the flowering time. A thorough review was conducted of 103 peer-reviewed research publications, which are listed in Table 1. Table 1 provides a comprehensive overview of genes with diverse functionalities that contribute to successful flowering time engineering. These genes include transcriptional regulators, non-coding RNAs, enzymes, signaling molecules, chromatin modifiers, epigenetic modifiers, and many more (Figure 4).

**Table 1:** Non-exhaustive overview of studies of flowering time using CRISPR.103 peer-reviewed research papers listed by the modified plant. In the table, “KO” represents knockouts, while “OE” represents overexpression. The terms “early”, “late”, or “No effect” indicate whether the time of flowering is earlier or later, or no difference compared to the wild type.

Author(s)	Year	Directed Gene(s)	Mechanism	Plant	Results in Flowering
Torre et al. [110]	2022	AaFRAT1	KO	Alpine cress	Early
Charrier et al. [111]	2019	MdTFL1.1, PcTFL1.1	KO	Apple, Pear	Early
Liu et al. [112]	2019	TFL1, AP1, SVP	KO	Arabidopsis	Abnormal flower development
Ning et al. [113]	2015	NACs	KO	Arabidopsis	Early
Nobusawa et al. [114]	2022	AMP1	KO	Arabidopsis	Early
Capovilla et al. [115]	2017	FLM-β	KO	Arabidopsis	Early
Branchat et al. [116]	2020	FDP, fd	KO	Arabidopsis	Early, late
Lian et al. [117]	2021	MIR172s	KO and OE	Arabidopsis	Early, late, no effect
Yan et al. [118]	2017	KHZ1 and KHZ2	KO and OE	Arabidopsis	Late and early
Hyun et al. [119]	2015	FT and SPL	KO	Arabidopsis	Late
Hou et al. [120]	2019	AtMIR396	KO	Arabidopsis	Late
Yao et al. [121]	2019	mir167a	KO	Arabidopsis	Late
Huang et al. [122]	2019	OsNCED5	OE	Arabidopsis	Late
Wang et al. [123]	2021	RBP45D	KO	Arabidopsis	Late
Zhao et al. [124]	2022	CIS1	KO	Arabidopsis	Late
Yang et al. [125]	2023	AtAGL79	KO	Arabidopsis	Late
Pyott et al. [126]	2016	eIF(iso)4E	KO	Arabidopsis	No effect
Soto et al. [127]	2022	FT2	KO	Aspen	No report
Qin et al. [128]	2019	FTL9	KO	Brachypodium	Late
Jeong et al. [92]	2019	BraFLC2, BraFLC3	KO	Cabbage	Early
Jung et al. [129]	2021	BrSOC1	KO	Cabbage	Early
Hong et al. [130]	2021	BrVRN1	KO	Cabbage	Late
Shin et al. [131]	2023	BrLFY	KO	Cabbage	Late
Lee et al. [132]	2023	BrFT1 and BrFT2	KO	Cabbage	Late
Shin et al. [91]	2022	AGL19s, AGL24s	KO	Cabbage	Late
Park et al. [133]	2019	GI	KO	Cabbage	No report
Bellec et al. [134]	2022	15 genes	KO	Camelina	Early
Jiang et al. [135]	2018	BnaSDG8.A, BnaSDG8.C	KO	Canola	Early
Sriboon et al. [99]	2020	BnaC03.TFL1	KO	Canola	Early
Guo et al. [136]	2022	BnaCOL9	KO	Canola	Early
Ahmar et al. [137]	2022	BnaSVPs	KO	Canola	Early
Zhou et al. [138]	2022	BnaSVP, BnaSEP1	KO	Canola	Early, no effect
Odipio et al. [139]	2018	TFL1-like	KO	Cassava	Early
Bull et al. [140]	2018	AtFT	Ectopic expression	Cassava	Early
Liu et al. [141]	2023	CiTFL1a, CiTFL1b	KO	Chrysantemum	Early
Huang et al. [142]	2017	ZmCCT9	KO	Corn	Early
Li et al. [143]	2020	ZmPHYC1, ZmPHYC2	KO	Corn	Early
Takahashi et al. [144]	2022	GtFT2	KO	Gentian	Late
Ying et al. [145]	2022	BdRFS	KO and OE	Brachypodium	Early, late
Sheng et al. [146]	2021	YSL3	KO	Brachypodium	Late
Herath et al. [147]	2022	AcBFT2	KO	Kiwi	No effect
Gasic et al. [148]	2019	AcCEN4, AcCEN	KO	Kiwi	Early
Varkonyi et al. [149]	2021	CEN, CEN4, SyGl	KO	Kiwi	Early
Choi et al. [10]	2022	SOC1	KO	Lettuce	Late
Singer et al. [150]	2021	MsSPL8	KO	Lucerne	Early
Galindo-Sotomonte et al. [151]	2023	MSAD_264347	KO	Lucerne	Late
Wolabu et al. [152]	2023	MsFTa1	KO	Lucerne	Late
Shibuya et al. [153]	2018	EPHEMERAL1	KO	Morning Glory	Delay in petal aging
Andre et al. [154]	2022	FT2b	OE	Populus	Early
Elorriaga et al. [155]	2018	PLFY, PAG1, PAG2	KO	Populus	No report
Lebedeva et al. [156]	2022	StLFY	KO	Potato	Late, non-flowering
Li et al. [157]	2017	Hd2, Hd4, Hd5	KO	Rice	Early
Brambilla et al. [158]	2017	hbf1, hbf2	KO	Rice	Early
Zhou et al. [159]	2018	Ghd8	KO	Rice	Early
Cui et al. [160]	2019	se14	KO	Rice	Early
Wang et al. [161]	2020	OsGhd7	KO	Rice	Early
Karthika et al. [162]	2021	MSH2	KO	Rice	Early
Leon et al. [163]	2021	OsGA20ox2	KO	Rice	Early
Sun et al. [164]	2022	qHD5	KO	Rice	Early
Yin et al. [165]	2023	HBP1	KO	Rice	Early
Sedeek et al. [166]	2023	Hd2, Hd4, Hd5	KO	Rice	Early
Guo et al. [167]	2022	OsFTL4	KO	Rice	Early
Sun et al. [168]	2021	OsLHY	KO	Rice	Early, late
Zhang et al. [169]	2020	OsCCTs	KO	Rice	Early, late, no effect
Cui Y et al. [170]	2021	14 genes	KO	Rice	Early, late, no effect
Yasui et al. [171]	2017	MADS3	KO	Rice	Early/Late
Wu et al. [172]	2020	Ehd1	KO	Rice	Late
Li et al. [173]	2021	OsLHY	KO	Rice	Late
Liu et al. [174]	2021	OsHd2	KO	Rice	Late
Zhang et al. [175]	2022	ga3ox-2	KO	Rice	Late
Xu et al. [176]	2023	OsLUX	KO	Rice	Late
Zhang et al. [177]	2022	ospil12-1 and ospil12-2	KO	Rice	Late
Andrade et al. [178]	2022	LUX, ELF3	KO	Rice	Non-flowering
Dai et al. [179]	2021	HbFT1-2, HbTFL1-3	KO	Rubber Tree	No report
Wang et al. [180]	2022	SiPHYC	KO	Setaria	Early
Zhu et al. [181]	2022	spp1	KO	Setaria	No effect
Char et al. [182]	2019	SbFT	KO	Sorghum	Late
Han et al. [183]	2019	E1	KO	Soy	Early
Wang et al. [184]	2020	Gmprr37	KO	Soy	Early
Wang et al. [185]	2020	GmNMHC5	OE	Soy	Early
Zhaobo Li et al. [186]	2021	LNK2	KO	Soy	Early
Wan et al. [187]	2022	E1	KO	Soy	Early
Zhai et al. [188]	2022	E1	KO and OE	Soy	Early, late
Cai et al. [189]	2018	GmFT2a	KO	Soy	Late
Wang et al. [190]	2019	GmLCLa1-4	KO	Soy	Late
Cong Li et al. [191]	2020	GmPRR3bH6	KO	Soy	Late
Chen et al. [192]	2020	GmAP1	KO	Soy	Late
Zhao et al. A [193]	2022	GmPHYAs	KO	Soy	Late
Schmidt et al. [194]	2020	NtFT5	KO	Tobacco	Non-flowering
Soyk et al. [195]	2017	SP5G	KO	Tomato	Early
Lemmon et al. [196]	2018	SP5G	KO	Tomato	Early
Li et al. [197]	2018	SP and SP5G	KO	Tomato	Early
Hu et al. [198]	2022	SlDOF9s	KO	Tomato	Early
Moreira et al. [199]	2022	SP3C	KO and OE	Tomato	Early, late
Xu et al. [200]	2016	S1BOP	KO	Tomato	No effect
Lin et al. [201]	2021	SlMIR172c, SlMIR172d	KO	Tomato	No report
Kwon et al. [202]	2019	SP5G, SP, SlER	KO	Tomato	Early
Gupta et al. [203]	2022	TaSPL13	KO	Wheat	Early
Sun et al. [204]	2023	TaTFL1-5	KO	Wheat	Early
Chen et al. [205]	2022	FT-D1	KO	Wheat	Late
Errum et al. [206]	2023	TaPpd	KO	Wheat	Late
Huiyun Liu et al. [207]	2020	TaAQ and TaDq	KO	Wheat	No report

**Figure 5:**
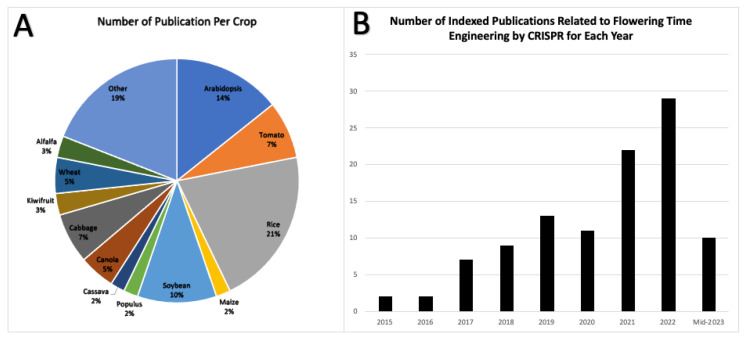
(**A**) Peer-reviewed publications on CRISPR-based flowering time engineering by crop (2015–mid-2023). (**B**) Annual number of indexed articles on CRISPR-mediated flowering time engineering. (**A**,**B**) are derived from the information presented in Table 1, which represents a statistical population that provides insight into the general state of the field.

The visual representation in Figure 5A shows the percentage distribution of plant species to which CRISPR technology has been applied. This distribution is likely influenced by the ease of transformation and use of model organisms. It is noteworthy that this approach is highly promising and can be extrapolated to various other commercial crops. Altering the flowering time provides a versatile means of addressing a wide range of cultivation, harvest, and post-harvest challenges.

The use of CRISPR technology for gene knockout, particularly in flowering projects, has seen sustained growth (Figure 5B). Further breakthroughs are expected as researchers refine and expand their understanding, leading to more sophisticated applications in the future.

## 5. Perspectives and Challenges of CRISPR-Mediated Flowering Time Engineering

Flowering-related breeding projects sometimes aim to completely inhibit flowering, while in other cases, a delay or advance of days to weeks is desired. To achieve these goals, the selection of the right pathway and protein to engineer is critical. In addition, the location of the mutation on the gene and the type of mutation affects the flowering time, which can vary from species to species based on the genotype or environmental factors.

About 300 genes are directly or indirectly involved in the flowering process [31]. To obtain the desired breeding results, it might be necessary to test several genes or gene combinations. However, the limited capacity of generating gene-edited plants slows down breeding. This slow pace hinders the exploration of many candidate genes as potential targets. In this regard, the need to increase the efficiency of transformation, regeneration, and gene editing processes is, therefore, essential to save time and explore a larger gene pool.

CRISPR technology is a leading innovation in plant breeding, offering precision, speed, and flexibility. The rapid generation of plants with desired traits surpasses the time limits of traditional breeding methods, while the scalability of CRISPR allows multiple target genes to be edited in parallel. However, to fully achieve CRISPR’s potential, it is increasingly recognized that it must be strategically combined with complementary techniques.

In this context, using inventive techniques and approaches to increase efficiency and save time is critical to the success of molecular breeding projects. Recent advances include improvements in delivery using improved Agrobacterium strains [208,209], viral delivery [210], improvements in regeneration using morphogenic regulators [211,212], and new approaches to use multiplex gene editing to study many genes simultaneously [213,214].

Through such comprehensive and interconnected approaches, breeders can gain new insights into the intricate web of regulatory networks that regulate the flowering time. This new knowledge can guide targeted breeding initiatives aimed at producing crop varieties with improved flowering traits.

## Data Availability

No new data were created or analyzed in this study. Data sharing is not applicable to this article.
